# Editorial: Sarcopenia and frailty: the role of physical activity for better aging

**DOI:** 10.3389/fpubh.2023.1303223

**Published:** 2023-11-10

**Authors:** Ricardo Aurélio Carvalho Sampaio, Priscila Yukari Sewo Sampaio, Luciane Portas Capelo, Marco Carlos Uchida, Hidenori Arai

**Affiliations:** ^1^Graduate Program in Physical Education, Federal University of Sergipe, São Cristóvão, Sergipe, Brazil; ^2^Department of Occupational Therapy, Federal University of Sergipe, Lagarto, Sergipe, Brazil; ^3^Institute of Science and Technology, Federal University of São Paulo, São José dos Campos, São Paulo, Sergipe, Brazil; ^4^Department of Adapted Physical Activity, School of Physical Education, University of Campinas, São Paulo, Sergipe, Brazil; ^5^National Center for Geriatrics and Gerontology, Obu, Aichi Prefecture, Japan

**Keywords:** aging, body composition, disability, muscle strength, physical function, public health

As the global population continues to age, the prevalence of age-related health conditions such as sarcopenia and frailty has become a significant concern for public health worldwide. These conditions affect the quality of life of older adults and place a substantial burden on healthcare systems. Especially because they increase the likelihood of falls, hospitalizations, multimorbidity, and functional disability (1, 2). In addition, sarcopenia and frailty are mortality indicators in both community-dwelling and institutionalized older adults (3).

In this Research Topic, we have assembled a collection of 10 research articles that delve into the complex relationship between sarcopenia, frailty, physical activity, and aging. These articles shed light on crucial aspects of these conditions and provide valuable insights for aging and public health, including those related to lifestyle factors for healthful and successful aging, chronic disease management across the life-course to promote healthy aging, and evidence-based programs and practices.

The article by Gomez-Campos et al. explored the relationship between age and handgrip strength (HGS), a key component of frailty and a predictor of future morbidity and mortality. Their research analyzed data from 5,376 Chilean participants (from 6 to 80 years old). They found that there is a non-linear relationship between chronological age and HGS from childhood to senescence. The proposed percentiles offer valuable insights into monitoring HGS, providing clinicians and researchers with percentiles by age and sex for assessing muscle strength levels.

Considering that low hemoglobin levels/anemia, osteoporosis, and sarcopenia are common in older people, two studies were conducted to verify such associations. Firstly, Liu Q. et al. investigated the correlation between hemoglobin levels and osteoporosis among older Chinese individuals. Their cross-sectional study had a sample of 1,068 individuals aged 55–85 years, and revealed a significant link between lower hemoglobin levels and bone mineral density, even after controlling for several confounders. This emphasizes the importance of considering both hematological and musculoskeletal factors in the assessment of frailty and bone health.

In another exploration of hemoglobin's role, Liu Y. et al. used data from the China Health and Retirement Longitudinal Study (CHARLS) to delve into the relationship between hemoglobin levels and sarcopenia in the Chinese population aged 60 and above (*n* = 3,055). Cross-sectionally, their findings highlighted a negative association between hemoglobin levels and sarcopenia, and low appendicular skeletal muscle mass adjusted by height. The cohort study data (*n* = 1,022) showed a negative association of hemoglobin level and low physical performance; with sarcopenia; and skeletal muscle mass.

Chen X. et al. addressed the critical issue of mortality in hemodialysis patients. They aimed to identify physical performance (i.e., gait speed, the Timed Up and Go, and the Short Physical Performance Battery) and muscle strength (i.e., HGS) as key predictors. The authors showed that muscle strength and physical performance, rather than muscle mass, predict all-cause mortality (*n* = 923 hemodialysis patients from China; 8.6% have died after a median of 14 months). These findings present the role of strength and physical performance rather than muscle mass in sarcopenia, as well as in hemodialysis patients, with implications for the care and management of this condition.

Supporting that physical exercise is an important intervention to promote health and fitness in sarcopenia, Xiang et al. conducted a bibliometric analysis of research on exercise interventions for this disease published between 2003 and 2022. Specifically, the United States of America is currently the country with the largest number of publications, and Alfonso Cruz-Jentoft, from Spain, first author of both European Consensus on the definition and diagnosis of sarcopenia (4, 5), the most cited author. Their study provided an overview of the current state of research and identified hotspots and trends in the field.

Takahashi et al. investigated the relationship between activity diversity (i.e., type, frequency, evenness) and frailty in a 2-year longitudinal study of Japanese community-dwelling older adults. A total of 207 non-frail participants at baseline were enrolled and 30.9% of them had incident frailty during the follow-up period. Their findings indicated that activity type and evenness were predictors of frailty after adjustments for sociodemographic and psychosomatic factors, underscoring the importance of considering not only the quantity but also the variety of activities in promoting healthy aging.

Ohta et al. highlighted the intricate interplay between physical and cognitive aspects of aging and emphasized the need for tailored interventions based on sex and age. They identified a stronger linear association of sarcopenia severity with poor cognitive function in women compared with men in their study. The study had a cross-sectional approach of regional cohorts of the Integrated Research Initiative for Living Well with Dementia (IRIDE) Cohort Study with 6,426 Japanese older people.

De lima et al. investigated, in a randomized 14-week interventional study, the effects of agility ladder training with a cognitive task in healthy older adults. This approach demonstrated the potential for improving both physical function and cognitive performance, through innovative interventions in aging populations considering sarcopenia and frailty.

Chen A. et al. conducted a comprehensive analysis of the relationship between body habitus and frailty in a community-based sample of 840 Chinese adults. Their research identified high body mass index (BMI) and waist-hip ratio (WHR) as significant risk factors for frailty, underpinning the importance of considering multiple body composition measures in assessing frailty risk.

Finally, Veen et al. highlighted the benefits of exceeding minimum activity guidelines to optimize performance in activities of daily living and overall functioning. They investigated the impact of accumulating twice the recommended minimum amount of moderate-to-vigorous physical activity (MVPA) on physical function (i.e., HGS, 5 times sit-to-stand, squat jump, and 6-min walk test) in older adults (*n* = 193). Their study revealed that individuals who engaged in more than 300 min of MVPA per week exhibited better physical function, particularly in walking performance.

Collectively, the articles on this Research Topic make an important step forward approach to sarcopenia, frailty, and the role of physical activity in aging populations, as well as underlying factors. As we continue to grapple with the implications of global aging, the insights gained from these studies may inform public health policies and interventions, inspire further research, and lead to strategies with the aim of mitigating sarcopenia and frailty in older adults, promoting healthy and wellbeing aging.

## Author contributions

RS: Writing—original draft, Writing—review and editing. PS: Writing—original draft, Writing—review and editing. LC: Writing—original draft, Writing—review and editing. MU: Writing—original draft, Writing—review and editing. HA: Writing—original draft, Writing—review and editing.
